# Assessing the effects of interprofessional education by hospital pharmacists on pharmaceutical students using a self-evaluation scale

**DOI:** 10.1186/s40780-024-00382-6

**Published:** 2024-10-01

**Authors:** Fuka Aizawa, Hirofumi Hamano, Naoto Okada, Kenta Yagi, Mitsuhiro Goda, Hideki Nawa, Yuya Horinouchi, Toshimi Nakamura, Harumasa Hakuno, Kazuaki Shinomiya, Yoshito Zamami, Masahiko Azuma, Masashi Akaike, Keisuke Ishizawa

**Affiliations:** 1 Department of Pharmacy, Tokushima University Hospital, 2-50-1 Kuramoto-Cho, Tokushima, 770-8503 Japan; 2 Department of Clinical Pharmacology and Therapeutics, Tokushima University Graduate School of Biomedical Sciences, 3-18-15 Kuramoto-Cho, Tokushima, 770-8503 Japan; 3 Department of Pharmacy, Okayama University Hospital, 2-5-1 Shikata-Cho Kita-Ku, Okayama, 770-8558 Japan; 4 Pharmacy Department, Yamaguchi University Hospital, 2-1-1 Minamikogushi, Ube, Yamaguchi 755-8505 Japan; 5 Clinical Research Center for Developmental Therapeutics, Tokushima University Hospital, 2-50-1 Kuramoto-Cho, Tokushima, 770-8503 Japan; 6 School of Pharmacy, Shujitsu University, 1-6-1 Nishikawahara Naka-Ku, Okayama, 703-8516 Japan; 7 Department of Pharmaceutical Care and Clinical Pharmacy, Tokushima Bunri University, Nishihama Yamashiro-Cho, Tokushima, 770-8514 Japan; 8 Department of Medical Education, Tokushima University Graduate School of Biomedical Sciences, 3-18-15 Kuramoto-Cho, Tokushima, 770-8503 Japan

**Keywords:** Interprofessional collaboration, Interprofessional education, Multidisciplinary education, Pharmaceutical training, Multidisciplinary team medicine

## Abstract

**Background:**

Understanding the roles and competencies of professions outside of one’s specialty is essential for providing efficient healthcare. However, it is difficult for medical professionals to understand the roles and competencies of other related professions while performing their duties. This study examined the impact of clinical practice-based interprofessional education (IPE) on pharmacy students, who are future medical professionals.

**Methods:**

Sixty-eight pharmaceutical students undergoing clinical practice were divided into non-IPE or IPE groups, with the IPE group attending an educational program with medical students conducted by doctors, pharmacists, and teachers during the clinical practice period. The effect was evaluated through a group survey using self-administered questionnaires focusing on contributing to multidisciplinary team medicine based on the Readiness for Interprofessional Learning Scale. The survey included specific behavioral objectives (SBOs), the Readiness for Interpersonal Learning Scale (RIPLS), and Kikuchi’s Scale of Social Skills (KiSS-18).

**Results:**

Regardless of group, SBOs [non-IPE: 3.2, 95% CI (2.6–3.8), *p* < 0.001; IPE: 3.7, 95% CI (2.5–4.9), *p* < 0.001] and social skills [non-IPE: 4.0, 95% CI (2.5–6.1), *p* < 0.001; IPE: 6.7 95% CI (3.0–10.4), *p* < 0.001] showed improvement after the clinical practice. In RIPLS Factor 3, pharmacy students with IPE awareness scored significantly higher by 1.5 points [95% CI (0.2–2.8), *p* = 0.025] post-practice than those without IPE awareness.

**Conclusions:**

This study suggests that IPE for students during clinical practice could enhance their expertise in multidisciplinary medicine and facilitate the development of seamless team care in the future.

**Trial registration:**

This study was retrospectively registered and conducted in compliance with the “Ethical Guidelines for Medical Research Involving Human Subjects” and was approved by The Ethics Committee of Tokushima University Hospital (approval number: 3544).

## Background

Medical professionals contribute to the healthcare system by utilizing expertise acquired during their university years. In recent years, collaboration among multidisciplinary professionals has become common owing to the worldwide promotion of team medicine [1]. However, the expertise required from medical professionals is becoming more complex in the context of advanced medical care [2]. For instance, pharmacists are expected to carry out interpersonal tasks, formulate drug therapy plans, and assess the potential therapeutic effects and side effects of these plans. In Japanese medical colleges, students acquire a comprehensive understanding of various fundamental disciplines such as physiology, pharmacology, chemistry, physics, pharmaceutical formulations, and ethics.

In addition, students undergo simulated patient care preparation for clinical practice. Although such knowledge is necessary for team medicine, the students have few opportunities to understand the importance of collaboration and how the knowledge they acquire during school is applicable in clinical situations. Furthermore, learning in the limited space of a university provides few opportunities to study in collaboration with other medical professionals and students. Further, there are few opportunities to experience collaboration among people from different professions. Understanding the duties and capabilities of related occupations is difficult, making role-sharing increasingly challenging. We considered that this difficulty can be addressed through interprofessional education (IPE), which allows students to learn about collaboration with other professionals [3] in clinical practice with medical professionals.

IPE has been incorporated into the curricula of several universities and has been studied from various perspectives [4–7]. Several scales have been used to assess IPE implementation in university education, such as the readiness for interprofessional learning scale (RIPLS) for readiness and orientation toward IPE [8] and Kikuchi’s scale of social skills (KiSS-18) for communication skills essential for multidisciplinary collaboration. For example, previous studies focusing on medical students (e.g., medical, dental, nurse, pharmacy, and clinical nutrition) have evaluated the impact of IPE on attitudes toward team medicine by conducting IPE and assessing the results with RIPLS [9–11]. Gifu Pharmaceutical University conducts IPE in collaboration with Gifu University and Heisei Medical College and evaluates its effects on pharmacy students using RIPLS and KiSS-18 [12]. In addition, Chiba University has been conducting IPE during hospital training of pharmacy students since 2007 and has reported their efforts [13].

Although the impact of IPE on students’ learning attitudes in universities is clear, there are limited opportunities to use today’s changing medical practice and patients as subjects to implement a medical plan with other student professionals that accurately consider clinical questions. In fact, few university employees work in hospitals, so students often learn about clinical practice in environments disconnected from actual clinical settings. Moreover, the extent to which the education provided by the universities benefits students in their clinical practice or their work as medical professionals remains unclear. Clinical practice helps students use the knowledge acquired at university and experience how they should behave as members of the medical profession when they meet patients or medical staff. Moreover, a hospital-based educational system may offer students opportunities to experience clinical care through team-based, student-led medical learning. Therefore, evaluating the efficacy of IPE in clinical practice with medical professionals would immensely affect the evolution of team medicine. Although the importance of IPE in clinical practice and the factors it influences have been acknowledged, the actual educational impact remains unclear [14, 15].

This study thus evaluates the impact of medical staff engaged in clinical work conducting IPE with students at Tokushima University, Japan, as part of their training. Specific behavioral objectives (SBOs) related to participation in team medicine from the 2013 revision of the Model Core Curriculum for Pharmaceutical Education, KiSS-18, and RIPLS were used to evaluate the difference in the assessment results before and after the training.

## Methods

### Study design and participants

This observational study evaluated the impact of IPE on pharmacy students in clinical practice. Participants completed self-administered questionnaires assessing the IPE program before and after a hospital internship. The questionnaires were distributed and collected by the faculty.

A total of 68 fifth-year pharmacy students at the University of Tokushima who underwent hospital practical training at the Tokushima University Hospital in the 2018–2019 academic year were included in the study. None of the students had any experience with IPE programs. Before inclusion, the purpose, methods, risks and benefits, protection of personal information, modification or discontinuation of the study, handling of research results, consent and withdrawal, and conflicts of interest regarding the study were explained to all participants. The study was conducted in compliance with the Ethical Guidelines for Medical Research Involving Human Subjects and with the approval of The Ethics Committee of Tokushima University Hospital (approval number: 3544). We informed the participants in writing that their participation in the study would not affect their academic performance and obtained their written consent.

The participants learned the required clinical training curriculum set by the government, that is, the knowledge and skills of the basic tasks required for hospital pharmacists and patient care, irrespective of consent. IPE was conducted with physicians and sixth-year medical students in the Department of Respiratory Collagen Disease, who provided their consent to participate. Therefore, 13 pharmacy students who accepted practical training in the Department of Respiratory and Collagen Diseases consented to participate in IPE. The remaining 55 students were non-IPEs, intervening in patient care that included collaboration with other healthcare professionals, both ward pharmacists and other healthcare professionals, to minimize the differences in educational content. The IPE program was implemented as part of students’ clinical training, with participants randomly assigned 1:1 to a pharmacist for practice. The random allocation was blinded, ensuring that the selection process was objective and not influenced by students’ preferences or motivations toward team-based medicine. This minimized the risk of selection bias, where highly motivated students might have preferentially joined the IPE group. Additionally, we reviewed the GPA of both groups and found no significant differences, further validating our results.

Although COVID-19 began to spread in 2019, it did not affect the facilities where this study was implemented, and all procedures were carried out in accordance with the research plan. Medical students enrolled in the Faculty of Medicine at Tokushima University during the clinical training period were included in the study. Three pharmacists, one physician in the Department of Respiratory Collagen Disease, and two Tokushima University Hospital faculty members were responsible for conducting the IPE.

### Contents of IPE

**Fig. 1:**
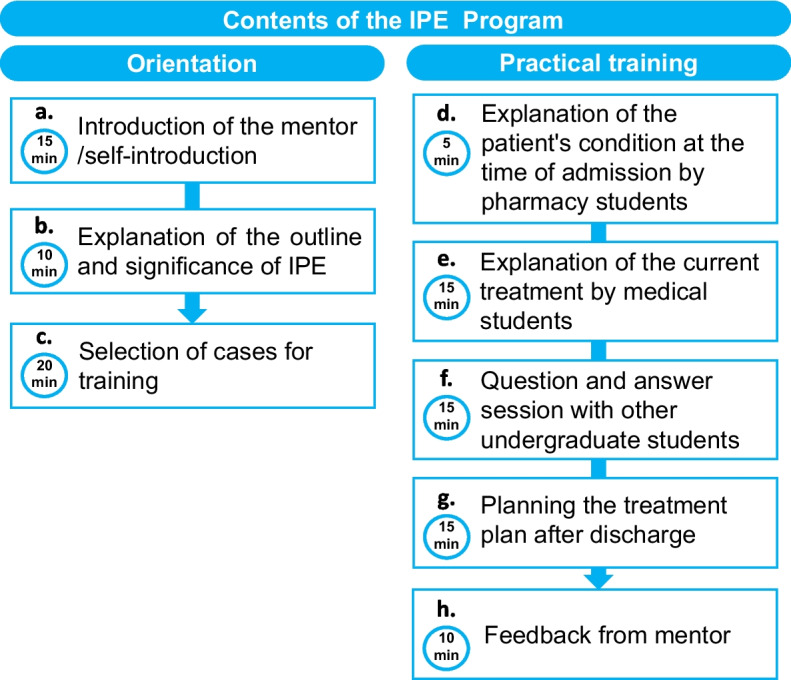
Clinical practice-based-IPE program. The plan implemented in this study is shown in the figure; the IPE program was developed over three orientation sessions and five training sessions

In IPE, a patient admitted to the Department of Respiratory and Collagen Diseases at Tokushima University Hospital during practical training was used as a case for planning medical care. At the time of admission, the patient consented to students’ participation in the medical procedures for the purpose of clinical education. A group of two to three pharmacy students and two to three medical students discussed the treatment and care for effective patient discharge and the post-discharge treatment and care for them and their families. The training was divided into two sessions—orientation and practical training—with around a week gap between them (Fig. 1). The orientation consisted of (a) an introduction of the mentor/self-introduction (15 min.), where the mentor and the students briefly introduced themselves; (b) an explanation of IPE outline and its significance (10 min.), where the mentor explained the day’s training schedule and the implication of IPE; and (c) selection of cases for training (20 min.), where the mentor and the students selected suitable cases for practice among the cases that the students were already in charge of. For the selected case, each student had to check the patient’s medical record and understand their condition. The practical training consisted of (d) an explanation of the patient’s condition at the time of admission by pharmacy students (5 min.), where they explained the details of the initial interview, such as the medication status and history that might affect the treatment; (e) an explanation of the current treatment by medical students (15 min.), where they explained the treatment progress before and after hospitalization, including diagnostic imaging; (f) a question-and-answer session with other undergraduate students (15 min.), where the students asked their queries; (g) development of a post-discharge treatment plan (15 min.), which identified and discussed the problems in home life and outpatient care; and (h) feedback from a mentor (10 min.), where teachers gave feedback to the group.

### Survey items

The survey items were SBOs, KiSS-18, and RIPLS. The survey was administered to the non-IPE and IPE groups before and after training. Questionnaire responses were obtained from all participating students for pre-education data after consent was obtained and for post-education data following IPE implementation. The contents of the team medicine SBOs, KiSS-18, and RIPLS are presented in Table 1. A five-point Likert scale was used for the SBOs, KiSS-18, and RIPLS. For RIPLS, the factors were Factor 1: Teamwork and collaboration; Factor 2: Understanding the need for IPE; and Factor 3: Professionalism in the profession [16].

**Table 1:** Survey items

SBOs (team medicine)	
1	To be able to explain the role and importance of pharmacists in team medicine
2	To be able to explain the purpose and composition of various medical teams and the roles of their members
3	To be able to explain the significance and specific methods of medical cooperation between hospitals and the community (collaborative clinical path, joint guidance at the time of discharge, hospital-pharmacy cooperation, cooperation with related facilities, etc.)
4	To be able to explain the significance and specific methods of cooperation between hospitals and the community (collaborative clinical path, joint guidance at the time of discharge, hospital-pharmacy cooperation, cooperation with related facilities, etc.)
**KiSS-18**	
1	Are you a person who is not easily distracted when talking with others?
2	Do you have the ability to tell others what you want them to do?
3	Do you do a good job of helping others?
4	Are you able to calm others down when they are upset?
5	Can you start a conversation with a stranger quickly?
6	Are you able to handle problems that arise with people around you?
7	When you feel scared or frightened, can you handle it well?
8	Can you reconcile with people with whom you have had an unpleasant experience?
9	Can you decide what to do and how to do it when you are working (studying)?
10	Do you feel comfortable participating in conversations with others?
11	Do you have the ability to handle situations where others take refuge?
12	Are you able to quickly identify problems in your work (or studies)?
13	Are you able to express your emotions and feelings honestly?
14	Are you able to handle conflicting stories from all over the place?
15	Are you able to introduce yourself well to new people?
16	Are you able to apologize immediately when you make a mistake?
17	Can you get along well with people around you even if they have ideas that differ yours?
18	Do you find it difficult to set goals for your work (or study)?
**RIPLS**	
1	Learning with other students will help me become a more effective member of a health care team
2	Patients would ultimately benefit if healthcare students worked together to solve patient problems
3	Shared learning with other healthcare students will increase my ability to understand clinical problems
4	Learning with healthcare students before qualification would improve relationships after qualification
5	Communication skills should be learned with other healthcare students
6	Shared learning will help me to think positively about other professionals
7	For small group learning to work, students need to trust and respect each other
8	Team-working skills are essential for all health care students to learn
9	Shared learning will help me understand my own limitations
10	I do not want to waste my time learning with other healthcare students
11	It is not necessary for undergraduate healthcare students to learn together
12	Skills for solving clinical problems can only be learned with students from my own department
13	Shared learning with other healthcare students will help me to communicate better with patients and other professionals
14	I would welcome the opportunity to work on small-group projects with other healthcare students
15	Shared learning will help to clarify the nature of patient problems
16	Shared learning before qualification will help me become a better team worker
17	The function of nurses and therapists is mainly to provide support for doctors
18	I am not sure what my professional role will be
19	I have to acquire much more knowledge and skills than other healthcare students

Factor 1 was categorized into items 1–9 and 13–16; Factor 2 into items 10 and 11; and Factor 3 into items 12 and 17–19. They were evaluated based on the total score of each category. Items 10–12 and 17–18 were reversed items.

### Statistical analysis

The questionnaires were collected by using students’ attendance numbers for identification. The information was anonymized after collection by setting a different identification number and using it for analysis. Only responses from participants who completed all survey items were included in the analysis. Incomplete or missing responses were excluded to ensure data reliability and validity. This exclusion criterion was applied uniformly across both the IPE and non-IPE groups to maintain the integrity of the comparisons made in the study. The pre- and post-practice ratings of the SBOs, KiSS-18, and RIPLS were compared. For each dependent variable (SBOs, KiSS-18, and RIPLS Factors 1–3), a linear mixed model analysis was conducted with the research participant as the variable factor, and the time factor (before and after the training), the group (non-IPE vs. IPE), the interaction term (group with time factor), and the value of the dependent variable before training as fixed factors. The effect size was evaluated by η^2^. The statistical significance level was set at 0.05 on both sides. The statistical software IBM SPSS Statistics v. 24.0 (IBM Corp., NY, USA) was used for analysis.

## Results

### Participant characteristics

A total of 66 participants of the 68 students who had undertaken practical training consented to participate in this study [consent rate: 97.1% (66/68)]. Of these, 53 participants in the non-IPE group and 13 in the IPE group were analyzed. The non-IPE group included 19 men and 34 women, whereas the IPE group included 3 men and 10 women.

### Effectiveness of the IPE program

The distribution of the continuous quantity data of SBOs, KiSS-18, and RIPLS Factors 1–3 is presented in Table 2. In both the groups, the SBO (non-IPE: 3.2, 95% CI: 2.6–3.8; IPE: 3.7, 95% CI: 2.5–4.9), KiSS-18 (non-IPE: 4.3, 95% CI: 2.5–6.1; IPE: 6.7, 95% CI: 3.0–10.4), and RIPLS Factor 1 (non-IPE: 2.1, 95% CI: 0.7–3.5; IPE: 4.8, 95% CI: 2.0–7.6) rating scales were significantly higher after practice (Table 3). RIPLS Factor 2 was not rated significantly differently before and after the training, regardless of IPE. RIPLS Factor 3 of the IPE group showed a similar scale in the non-IPE group, whereas a significant increase was detected in the former (*p* = 0.004). In RIPLS Factor 3, there was a 1.5 (95% CI: 0.2–2.8) point increase in the amount of change before and after the training between the IPE and non-IPE groups (Table 4). This result indicates a significant intervention effect of IPE for RIPLS Factor 3 (*p* = 0.025, η^2^ = 0.08).

**Table 2:** Confirmation of the distribution of continuous quantity data

	Skewness	Kurtosis
SBOs_pre	-0.266	0.459
KiSS-18_pre	0.503	-0.064
RIPLS_Factor1_pre	0.581	-0.373
RIPLS_Factor2_pre	-0.712	0.391
RIPLS_Factor3_pre	0.711	1.580

**Table 3:** Effect of hospital practice on each item

	Non-IPE (*n* = 53)			IPE (*n* = 13)		
	LS mean	95% CI (lower limit, upper limit)	*P*-value	LS mean	95% CI (lower limit, upper limit)	*P*-value
SBOs						
Before practice	12.7	(12.3—13.1)	-	12.6	(11.8—13.3)	-
After practice	15.9	(15.5—16.3)	-	16.3	(15.5—17.0)	-
Discrepancy (after—before)	3.2	(2.6—3.8)	** < 0.001**	3.7	(2.5—4.9)	** < 0.001**
KiSS-18						
Before practice	58.6	(57.3—59.8)	-	58.5	(55.9—61.0)	-
After practice	62.9	(61.6—64.1)	-	65.2	(62.6—67.7)	-
Discrepancy (after—before)	4.3	(2.5—6.1)	** < 0.001**	6.7	(3.0—10.4)	** < 0.001**
RIPLS_Factor1						
Before practice	53.4	(52.4—54.3)	-	53.6	(51.7—55.5)	-
After practice	55.5	(54.6—56.5)	-	58.4	(56.5—60.3)	-
Discrepancy (after—before)	2.1	(0.7—3.5)	**0.003**	4.8	(2.0—7.6)	**0.001**
RIPLS_Factor2						
Before practice	8.5	(8.2—8.8)	-	8.6	(8.0—9.2)	-
After practice	8.4	(8.1—8.7)	-	9.2	(8.6—9.8)	-
Discrepancy (after—before)	-0.1	(-0.5—0.3)	0.621	0.6	(-0.2—1.5)	0.159
RIPLS_Factor3						
Before practice	14.1	(13.7—14.5)	-	14.0	(13.2—14.7)	-
After practice	14.3	(13.9—14.7)	-	15.7	(14.9—16.5)	-
Discrepancy (after—before)	0.2	(-0.3—0.8)	0.402	1.8	(0.6—3.0)	**0.004**

*LS mean* Least squares mean; *95% CI* 95% confidence interval

*P*-value: test for the time factor in a linear mixed model

**Table 4:** Difference between the two groups (IPE-taking—IPE-not-taking)

	LS mean	95% CI (lower limit, upper limit)	*P*-value	Effect size: η^2^
SBOs	0.5	(-0.9—1.9)	0.484	0.01
KiSS-18	2.4	(-1.7—6.5)	0.250	0.02
RIPLS_Factor1	2.6	(-0.5—5.8)	0.098	0.04
RIPLS_Factor2	0.7	(-0.2—1.7)	0.139	0.03
RIPLS_Factor3	1.5	(0.2—2.8)	**0.025**	0.08

*LS mean* Least squares mean, *95% CI*: 95% confidence interval

*P*-value: test for the interaction term (time factor* group) in a linear mixed model

## Discussion

First, we analyzed the skewness and kurtosis values presented in Table 2 and found both to be within the generally accepted range of ± 2, indicating a normal distribution. This suggests that the data distribution does not significantly deviate from normality, allowing us to confidently use parametric tests to interpret the results. Given the nature of self-reported questionnaire data, slight variations in skewness and kurtosis were expected and factored into our analysis. After evaluating the effectiveness of practical IPE training for pharmaceutical students by hospital pharmacists, students were found to differ in their attitudes toward professionalism before and after the training. Regardless of whether the students received IPE, their understanding of the content of the SBOs, that is, the “attitudes necessary for the practice of team medicine,” improved because the self-evaluation of the team medicine SBOs increased after training owing to clinical experience. In addition, as the results for KiSS-18 show, gaining new relationships through practical training led to improved social skills. RIPLS evaluation showed that the understanding of teamwork, collaboration, and the need for IPE was improved through ward practice. Hence, the students recognized the importance of IPE education, including those who did not receive IPE.

The increase of IPE training specificity was crucial to professionalism. To understand the expertise of other professions, ward practice without IPE was insufficient. However, IPE improved the evaluation of relevant items. Since ward practice focused only on self-territory occupation, the evaluation of relevant items may have increased through actual discussions with various professionals during IPE. Students without these experiences, including pharmacy and medical students, are typically aware of their counterparts as professionals; however, they lack an understanding of each other’s roles and abilities [17].

Hospital practical training involving IPE helps undergraduates understand the importance of specific professions and team medicine. Although hospital practice allows pharmacy students to cooperate with multiple professionals, pharmacy students with no clinical experience have limited opportunities to engage with other professionals during practice. Therefore, IPE programs are designed at each facility and vary in terms of content and duration [18]. This study was conducted during two days of clinical training. Renschler et al. investigated the short- and long-term effects of joint learning on teamwork and changes in attitudes toward professionalism among medical students [19]. Their results suggested that the alteration of students’ attitudes toward team medicine is not affected by internship duration. This study suggests that IPE may help overcome this problem, even if the intervention is short.

There are some limitations to this study. First, there is a difference in the number of participants between the two groups. While it would be preferable to have similar numbers of participants in each group, medical and pharmacy students require different clinical training programs, and it takes time to establish a cross-faculty learning system; as such, the IPE was limited to 13 students, the maximum number that could be recruited. We are planning a joint clinical practice program among faculty. Second, because the evaluation was conducted for around 11 weeks, the educational effect on long-term behavioral changes could not be measured. Thus, while short-term improvements in social skills and interprofessional collaboration were observed, the long-term impact remains unclear. Factors other than practical training could influence awareness and behavioral change over a longer evaluation period. Hence, this analysis was conducted before and after hospital training to eliminate the effects of factors other than ward practice. Nguyen et al. conducted an IPE program for students of medicine, oral and orofacial medicine, preventive medicine, traditional Vietnamese medicine, pharmacy, nursing, and midwifery students [20]. The program included two lectures and eight practical sessions, covering a broader range of disciplines and a longer duration than our study. All disciplines showed a significant increase in RIPLS scores following IPE. Although a direct comparison with our study is challenging due to differences in the study design, the mean total RIPLS score increased by 8 points after the intervention.

Correa et al. examined the additional effects of active learning and lectures on group-based IPE [21]. Their program included 4 h per week over 12 weeks, consisting of 2 h of classroom instruction and 2 h of home study, a longer period than our setting. Although there were no significant differences in knowledge between the two groups, active learning tended to improve teamwork abilities. They also assessed knowledge retention over a 6-month period and found. No correlation between knowledge mastery and time spent, reporting that shorter, intermittent learning sessions are more likely to promote long-term retention than longer continuous learning periods [22]. The WHO describes IPE as occurring when students from two or more professions learn about, from, and with each other to facilitate effective collaboration and improve health outcomes [23]. It also highlights IPE as a preparatory step for health care professionals to address community health care needs. This suggests that our program might benefit from diversifying participants and incorporating intermittent and ongoing learning opportunities to enhance the educational experience. Finally, in hospitals, different conditions (e.g., patient status, such as acute or chronic) change the factors necessary for medical staff to communicate with each other [24, 25]. Hence, the learning effects found in this study need to be confirmed for other diseases and over a longer study period. To ensure the results were not affected by translating the scale from English to Japanese, the questionnaire was back-translated. Moreover, each scale used in the study has international recognition and holds potential for global applicability, as demonstrated in this study.

## Conclusions

Collaboration with multiple professionals is essential to coping with the ever-changing complexity of healthcare. Patient satisfaction is influenced by team medicine [26]. To increase the number of medical professionals with practical skills, it is important to consider how to develop personnel who can respect others and find ways to assist them from the early stages of their careers. This requires theoretical and practical learning in clinical situations. In addition, students could observe and experience the behavior of medical staff in a team environment during various medical situations in hospital setting. We predict that medical staff conducting IPE training will be allowed to objectively reconsider their present teams. If medical professionals regularly reviewing and revising their team medicine practices would positively influence the medical community and the educational effect on students. Further studies of other professionals, such as nurses, nutritionists, and clinical laboratory technicians, may provide more insight into practical education. The results suggest that the clinical practice-based IPE is effective in helping students learn more realistic team medicine as they observe the decision-making processes of other professionals and how pharmacists intervene with patients and medical staff.

## Data Availability

All data obtained and analyzed by the present study are included in this manuscript and not published elsewhere.

## Abbreviations

IPE: Interprofessional education
KiSS-18: 18 Items of Kikuchi’s scale of social skills
RIPLS: Readiness for interprofessional learning scale
SBO: Specific behavioral objective

## References

[1] Babiker A, El Husseini M, Al Nemri A, Al Frayh A, Al Juryyan N, Faki MO, et al. Health care professional development: Working as a team to improve patient care. Sudan J Paediatr. 2014;14:9–16. 27493399 PMC4949805

[2] Frenk J, Chen L, Bhutta ZA, Cohen J, Crisp N, Evans T, et al. Health professionals for a new century: transforming education to strengthen health systems in an interdependent world. The Lancet. 2010;376:1923–58. 10.1016/S0140-6736(10)61854-5. 10.1016/S0140-6736(10)61854-5 21112623

[3] Van Diggele C, Roberts C, Burgess A, Mellis C. Interprofessional education: tips for design and implementation. BMC Med Educ. 2020;20. 10.1186/s12909-020-02286-z. 10.1186/s12909-020-02286-z PMC7712597 33272300

[4] Abu-Rish E, Kim S, Choe L, Varpio L, Malik E, White AA, et al. Current trends in interprofessional education of health sciences students: a literature review. J Interprof Care. 2012;26:444–51. 10.3109/13561820.2012.715604. 22924872 10.3109/13561820.2012.715604 PMC7594101

[5] Cooper H, Carlisle C, Gibbs T, Watkins C. Developing an evidence base for interdisciplinary learning: a systematic review. J Adv Nurs. 2001;35:228–37. 10.1046/j.1365-2648.2001.01840.x. 11442702 10.1046/j.1365-2648.2001.01840.x

[6] Kent F, Keating JL. Interprofessional education in primary health care for entry level students–A systematic literature review. Nurse Educ Today. 2015;35:1221–31. 10.1016/j.nedt.2015.05.005. 26043657 10.1016/j.nedt.2015.05.005

[7] Kent F, Hayes J, Glass S, Rees CE. Pre-registration interprofessional clinical education in the workplace: a realist review. Med Educ. 2017;51:903–17. 10.1111/medu.13346. 28612407 10.1111/medu.13346

[8] Parsell G, Bligh J. The development of a questionnaire to assess the readiness of health care students for interprofessional learning (RIPLS). Med Educ. 1999;33:95–100. 10.1046/j.1365-2923.1999.00298.x. 10211258 10.1046/j.1365-2923.1999.00298.x

[9] Almazrou S, Alaujan S. Knowledge and Readiness for Interprofessional Learning Among Pharmacy and Clinical Nutrition Students at King Saud University. J Multidiscip Healthc. 2022;15:1965–70. 10.2147/JMDH.S360608. 36090649 10.2147/JMDH.S360608 PMC9462515

[10] Khalafi A, Sarvi Sarmeydani N, Akhoondzadeh R. Simulation-based Interprofessional Education (IPE) for Enhanced Attitude and Teamwork of Anesthesiology Residents and Nurse Anesthesia Students in Iran. J Adv Med Educ Prof. 2023;11:105–12. 10.30476/JAMP.2022.95701.1657. 37113681 10.30476/JAMP.2022.95701.1657 PMC10126715

[11] Lie DA, Fung CC, Trial J, Lohenry K. A comparison of two scales for assessing health professional students’ attitude toward interprofessional learning. Med Educ Online. 2013;18:21885. 10.3402/meo.v18i0.21885. 24300749 10.3402/meo.v18i0.21885 PMC3849511

[12] Yoko I, Takuya M, Tomoya T, Yoshihiro N, Hitomi T. Effect of Multidisciplinary Medical Care Team Education on Pharmacy Students - Short-term Effect Focused on RIPLS (Readiness for Interprofessional Learning Scale) and IEPS (Interdisciplinary Education Perception Scale). Japanese J Pharm Health Care Sci. 2018;44:191–202.

[13] Okubo M. Clinical interprofessional education implemented as part of practicaltraining: Participation by undergraduate students and its influence on them. Japanese J Pharma Educ. 2019;3:1–4.

[14] Ganotice F, Zheng B, Ng PY, Leung SC, Barrett EA, Chan HYC, et al. Towards a global partnership model in interprofessional education for cross-sector problem-solving. BMC Med Educ. 2023;23. 10.1186/s12909-023-04290-5. 10.1186/s12909-023-04290-5 PMC10283208 37340427

[15] O’Leary N, Salmon N, Clifford AM. ‘It benefits patient care’: the value of practice-based IPE in healthcare curriculums. BMC Med Educ. 2020;20. 10.1186/s12909-020-02356-2. 10.1186/s12909-020-02356-2 PMC7658912 33183276

[16] Tamura Y, Seki K, Usami M, Taku S, Bontje P, Ando H, et al. Cultural adaptation and validating a Japanese version of the readiness for interprofessional learning scale (RIPLS). J Interprof Care. 2012;26:56–63. 10.3109/13561820.2011.595848. 22233369 10.3109/13561820.2011.595848

[17] Hickey EL, Dumke EK, Ballentine RL, Brown BL. Prospective health students’ perceptions of the pharmacist role in the interprofessional team. J Interprof Care. 2018;32:250–3. 10.1080/13561820.2017.1381671. 29058498 10.1080/13561820.2017.1381671

[18] Arruzza E, Chau M, Hayre C. Interprofessional education (IPE) in medical radiation science: A scoping review. Radiography (Lond). 2023;29:398–407. 10.1016/j.radi.2023.01.021. 36780794 10.1016/j.radi.2023.01.021

[19] Renschler L, Rhodes D, Cox C. Effect of interprofessional clinical education programme length on students’ attitudes towards teamwork. J Interprof Care. 2016;30:338–46. 10.3109/13561820.2016.1144582. 27152538 10.3109/13561820.2016.1144582

[20] Nguyen HTT, Wens J, Tsakitzidis G, Valcke M, Nguyen HT, Duong TQ, et al. A study of the impact of an interprofessional education module in Vietnam on students’ readiness and competencies. PLoS ONE. 2024;19: e0296759. 10.1371/journal.pone.0296759. 38354173 10.1371/journal.pone.0296759 PMC10866504

[21] Correa CPS, Lucchetti ALG, da Silva EO, Lucchetti G. Short and medium-term effects of different teaching strategies for interprofessional education in health professional students: A randomized controlled trial. Nurse Educ Today. 2022;117: 105496. 10.1016/j.nedt.2022.105496. 35914346 10.1016/j.nedt.2022.105496

[22] Cepeda NJ, Pashler H, Vul E, Wixted JT, Rohrer D. Distributed practice in verbal recall tasks: A review and quantitative synthesis. Psychol Bull. 2006;132:354–80. 10.1037/0033-2909.132.3.354. 16719566 10.1037/0033-2909.132.3.354

[23] World Health Organization. "Framework for Action on Interprofessional Education & Collaborative Practice" 2010. https://www.who.int/publications/i/item/framework-for-action-on-interprofessional-education-collaborative-practice. Accessed 10 Sept 2024.

[24] Hood K, Cross WM, Cant R. Evaluation of interprofessional student teams in the emergency department: opportunities and challenges. BMC Med Educ. 2022;22. 10.1186/s12909-022-03954-y. 10.1186/s12909-022-03954-y PMC9764718 36536393

[25] Saunders R, Dugmore H, Seaman K, Singer R, Lake F. Interprofessional learning in ambulatory care. Clin Teach. 2019;16:41–6. 10.1111/tct.12764. 29436114 10.1111/tct.12764

[26] Meterko M, Mohr DC, Young GJ. Teamwork culture and patient satisfaction in hospitals. Med Care. 2004;42:492–8. 10.1097/01.mlr.0000124389.58422.b2. 15083111 10.1097/01.mlr.0000124389.58422.b2

